# Persistent lymphocytopenia in convalescent patients with COVID-19: dysregulated B cell, CD4^+^ T cell, and treg compartments in 7–12% of moderate-severe cases

**DOI:** 10.3389/fcimb.2025.1693743

**Published:** 2025-10-09

**Authors:** Hui An, Tongtong Yu, Anlan Wang, Hao Hu, Chen Zhang, Yongyu Wang, Ming Li

**Affiliations:** ^1^ School of Basic Medical Science, Wenzhou Medical University, Wenzhou, Zhejiang, China; ^2^ Department of Anesthesia and Critical Care, The Second Affiliated Hospital and Yuying Children’s Hospital of Wenzhou Medical University, Wenzhou, Zhejiang, China; ^3^ Key Laboratory of Pediatric Anesthesiology, Ministry of Education, Wenzhou Medical University, Wenzhou, Zhejiang, China

**Keywords:** COVID-19, convalescent patients, persistent lymphocytopenia, B cells, T cells, regulatory T cells

## Abstract

**Background:**

Long COVID manifests with heterogeneous clinical outcomes, potentially linked to immune dysfunction. However, the recovery of immune-cell subsets during convalescence remains incompletely understood.

**Methods:**

In this longitudinal cohort, 279 unvaccinated patients with confirmed SARS-CoV-2 infection (13 mild, 218 moderate, 48 severe) were enrolled. Peripheral lymphocyte subsets were analyzed by flow cytometry at admission and at 50 days post-symptom onset (DPSO 50).

**Results:**

Total T-cell counts normalized in 90–98% of patients in the moderate and severe groups by DPSO 50. Nevertheless, a subgroup exhibited persistent B-cell lymphopenia (<90 cells/µL) in 7.3% of moderate cases (median 77.1 cells/µL, IQR 51.9–83.8) and 12.5% of seltvere cases (median 54.5 cells/µL, IQR 28.4–79.3). Patients with B-cell deficiency also showed concurrent reductions in total T cells, CD4^+^ T cells, and CD4^+^CD25^+^CD127low/FOXP3^+^ regulatory T cells (Tregs). In moderate cases, CD4^+^ T cell and Treg counts correlated positively (r = 0.72, p < 0.001), independent of B-cell status, whereas this relationship was absent in severe cases, indicating severity-dependent immune dysregulation.

**Conclusions:**

Approximately 7–12% of moderate-to-severe COVID-19 survivors displayed persistent lymphopenia affecting B cells, CD4^+^ T cells, and Tregs at ~50 days post-symptom onset. These findings highlight distinct recovery trajectories and provide insights into Long COVID pathogenesis that may inform therapeutic strategies.

## Introduction

Post-COVID-19 conditions (PCC), affecting approximately 10–30% of individuals infected with SARS-CoV-2 (Altmann et al., 2023; Rudolph et al., 2025), are proposed to arise from unresolved immune system dysfunction. While acute-phase immune perturbations—including diminished cytotoxic CD8+ T and natural killer (NK) cells alongside compensatory expansions of double-negative T cells (DN T cells) and regulatory T cells (Tregs)—are extensively documented (Chen et al., 2020; Guan et al., 2020; Huang et al., 2020; Jin S. et al., 2021; Zahran et al., 2021; An et al., 2022), their resolution during convalescence remains contentious. Disease progression further complicates this trajectory: moderate-to-severe cases may initially elevate Treg concentrations as the body attempts to suppress hyperinflammation (Alsalman et al., 2022), yet critically ill patients often exhibit Treg depletion, exacerbating immune hyperactivation, inflammation, and clinical deterioration (Votto et al., 2023).

Immune abnormalities persist beyond acute illness: SARS-CoV-2-specific memory B and T cell responses remain detectable for up to 8 months post-infection (Sherina et al., 2021), though B-cell counts show conflicting trends—either increasing or decreasing at 2–8 months post-infection (Orologas-Stavrou et al., 2020; Kostopoulos et al., 2021; Ryan et al., 2022) —likely due to population heterogeneity (e.g., vaccination status) and variable follow-up intervals (Shuwa et al., 2021; Yang et al., 2021; Kudryavtsev et al., 2022).

To isolate SARS-CoV-2 intrinsic immunopathological effects, our study examines unvaccinated patients, excluding confounding vaccine-induced immune modulation. This cohort enables analysis of the virus impact on B/T cell dynamics, aiming to establish baseline recovery patterns critical for distinguishing virus-driven mechanisms from vaccine-modulated responses in future PCC research. Here, we provide a combined longitudinal analysis of B cells, CD4^+^ T cells, and Tregs up to 50 days post-symptom onset, a timepoint less commonly covered in existing literature. Many prior studies have focused on shorter follow-up periods or vaccinated cohorts, which may mask true virus-induced immune dysregulation. Our findings reveal a severity-dependent disruption in Treg coordination, which has not been extensively reported and may underlie heterogeneous Long COVID outcomes. Such insights are foundational for developing targeted therapeutic strategies to address persistent immune dysregulation in post-acute sequelae.

## Methods

### Ethics approval and consent to participate

This study was ethically approved by the Institutional Review Board (IRB) of Wenzhou Medical University (Approval Number: 2020002), and all research activities strictly complied with the ethical guidelines established in the 1975 Declaration of Helsinki. Prior to participation, written informed consent was obtained from all study subjects, ensuring transparency regarding sample collection procedures and data usage. The protocol adhered to international standards for human subject research, with ethical oversight maintained throughout the study duration.

### Study design

This longitudinal cohort study enrolled 279 unvaccinated individuals who had recovered from SARS-CoV-2 infection, drawn from a larger cohort of 685 confirmed COVID-19 patients admitted to 12 hospitals across Wenzhou City, Zhejiang Province, China between January 17 and March 20, 2020. Upon admission, patients were initially categorized into four clinical severity groups based on symptomatology and imaging criteria: (1) mild cases (symptomatic but no chest CT abnormalities), (2) moderate cases (fever, respiratory symptoms, and CT-confirmed pneumonia), (3) severe cases (respiratory distress with SpO_2_ ≤93% at rest, respiratory rate ≥30/min, or PaO_2_/FiO_2_ ≤300 mmHg), and (4) critical cases (mechanical ventilation, shock, multi-organ failure, or ICU admission). Discharge criteria required resolution of fever and respiratory symptoms for ≥3 days, radiological improvement on CT scans, and two consecutive negative RT-qPCR assays for SARS-CoV-2 RNA.

**Figure 1:**
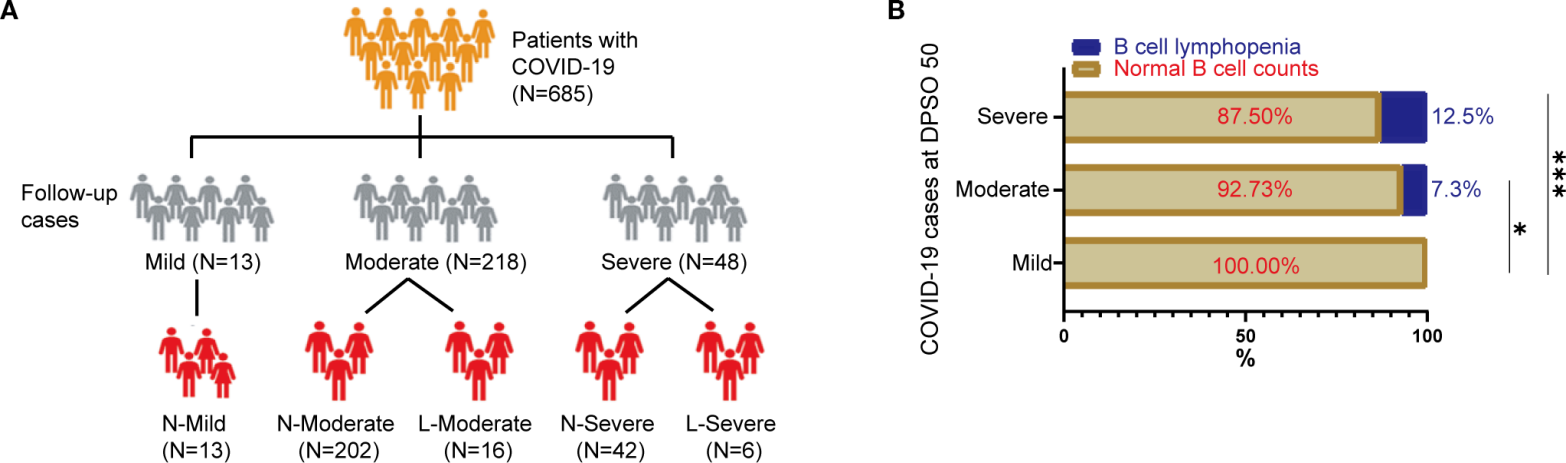
Study enrollment flowchart and immunophenotypic profiling of B-cell depletion in COVID-19 cohort. **(A)** Patient Enrollment and Stratification Process: This flowchart outlines the sequential inclusion of participants, highlighting eligibility criteria and allocation into severity-based subgroups (mild, moderate, severe) according to clinical manifestations and laboratory parameters. **(B)** B-Cell Lymphopenia Distribution by Disease Severity: This panel compares the prevalence of B-cell lymphopenia (<90 cells/μL) across disease severity subgroups. Patients were stratified as follows: N-Mild: Mild cases with normal B-cell counts (≥90 cells/μL). N-Moderate: Moderate cases with preserved B-cell levels. L-Moderate: Moderate cases exhibiting B-cell lymphopenia. N-Severe: Severe cases with normal B-cell counts. L-Severe: Severe cases with B-cell lymphopenia. Statistical analysis was performed using Chi-square test. Statistical significance is denoted by *p<0.05, ***p<0.001.

For this follow-up analysis, participants were stratified into three severity-based groups at enrollment: mild (n=13), moderate (n=218), and severe (n=48) (**Figure 1A**). During convalescence, a subset of patients exhibited persistent B-cell lymphopenia (B-cell count <90 cells/µL), a threshold aligned with established immunological benchmarks (Kam et al., 1996; Apoil et al., 2017; Jin XH. et al., 2021). To further investigate this phenomenon, participants were subsequently divided into four combined severity-B-cell status cohorts: 1) moderate cases with normal B-cell counts (NMo), 2) moderate cases with B-cell lymphopenia (LMo, <90 cells/µl) (Kam et al., 1996; Apoil et al., 2017; Jin XH. et al., 2021), 3) severe cases with normal B-cell counts (NS), and 4) severe cases with B-cell lymphopenia (LS) (**Figure 1B**). This dual-stratification enabled evaluation of disease severity and immune recovery heterogeneity in post-acute SARS-CoV-2 infection.

### Data collection and laboratory procedures

Comprehensive clinical and demographic data were systematically gathered using a standardized form adapted from the WHO/International Severe Acute Respiratory and Emerging Infection Consortium (ISARIC) protocol for severe respiratory infections. This dataset included electronic medical records, epidemiological histories, demographic profiles, clinical symptoms, laboratory test results, treatment protocols, and patient outcomes. Peripheral blood samples collected in ethylenediaminetetraacetic acid (EDTA)-anticoagulant tubes were obtained from participants at two critical timepoints: (1) during hospitalization for acute SARS-CoV-2 infection and (2) at 30-day post-discharge follow-up. Routine hematological assessments were performed to evaluate complete blood counts (CBC), including detailed quantification of white blood cells (WBC), neutrophils, lymphocytes, T cells, B cells, and natural killer (NK) cells. These analyses provided critical immunophenotypic and inflammatory biomarker data for longitudinal immune trajectory evaluation.

Exclusion criteria were rigorously applied to ensure data integrity, as outlined in **Figure 1A**. Patients diagnosed with confirmed COVID-19 were excluded from the study cohort if written informed consent was not obtained, thereby maintaining adherence to ethical and methodological standards. This dual-phase sampling strategy enabled characterization of both acute-phase immune dysregulation and post-acute immune recovery patterns, with laboratory protocols standardized across all participating institutions to minimize inter-hospital variability.

### Flow cytometric analysis

Peripheral blood samples (2 mL) were collected via venipuncture into ethylenediaminetetraacetic acid (EDTA)-anticoagulant tubes from confirmed SARS-CoV-2 patients at two timepoints: 1) during initial hospitalization prior to any therapeutic intervention, and 2) at the 30-day follow-up after hospital discharge. All samples were processed within 24 hours of collection. These samples were used to characterize lymphocyte phenotypes, including CD4^+^ T cells, CD8^+^ T cells, CD19^+^ B cells, natural killer (NK) cells, DN T cells (CD3^+^CD4^−^CD8^−^), and regulatory T cells (Tregs; CD4^+^CD25^+^CD127low/FOXP3^+^), following established protocols (Jiang et al., 2020). Phenotypic analysis was performed using a panel of fluorophore-conjugated monoclonal antibodies targeting key surface markers and intracellular transcription factors. Antibodies were sourced from BD Biosciences (San Jose, CA, USA) and BioLegend (San Diego, CA, USA), including: CD3^+^ T cell gating: PerCP-conjugated anti-CD3 (BD); CD4^+^ T cells: APC-conjugated anti-CD4 (BD); CD8^+^ T cells: APC/Cy7-conjugated anti-CD8 (BioLegend); Tregs: PE-Cy7 anti-CD25, FITC anti-CD127, and PE anti-FOXP3 (BD); B cells: APC-conjugated anti-CD19 (BD); and NK cells: APC anti-CD16 and BV510-conjugated anti-CD56 (BioLegend). The BD FACS Canto II flow cytometer (BD Biosciences) was employed for multicolor fluorescence detection. Lymphocyte subsets were identified using standardized gating strategies: CD4^+^ T cells: CD3^+^CD4^+^ population; CD8^+^ T cells: CD3^+^CD8^+^ population; DN T cells: CD3^+^CD4^−^CD8^−^ subset; Tregs: CD4^+^CD25^+^CD127low/FOXP3^+^ cells; B cells: CD3^−^CD19^+^ population; and NK cells: CD3^−^CD16^+^/CD56^+^ cells. This dual-timepoint approach enabled longitudinal assessment of immune reconstitution dynamics, with rigorous standardization across all experimental procedures to ensure reproducibility.

### Statistical analysis

Data are presented as medians with interquartile ranges (IQR) or frequencies with percentages, depending on variable distribution. The D’Agostino-Pearson omnibus normality test was applied to assess distribution normality. For parameters without available normal control data, reference ranges were derived from age- and sex-matched healthy individuals from our hospital’s clinical databases, and patient values were categorized as normal or abnormal relative to these thresholds.

Quantitative variables exhibiting non-normal distributions (e.g., lymphocyte counts, B cells, NK cells, CD4^+^/CD8^+^ T cells) were analyzed using non-parametric methods. A two-way non-parametric ANOVA (Scheirer-Ray-Hare test) was first applied to evaluate main effects and interactions, followed by *post-hoc* Kruskal-Wallis multiple comparisons with Dunn’s correction for pairwise contrasts. Categorical variables (e.g., sex, age group) were compared using Chi-square tests or Fisher’s exact test (when cell counts <5). Spearman’s rank correlation coefficients were calculated to assess associations between regulatory T cell (Treg) counts and B cell (CD19^+^) populations. Statistical significance was defined as p < 0.05, with all analyses performed using SPSS version 25.0 (IBM SPSS Statistics, Chicago, IL, USA).

## Results

### Clinical evaluation of patients

The study cohort comprised 279 patients (149 males, 130 females) with a median age of 48 years (interquartile range [IQR] 39–56). Age exhibited a significant association with disease severity (p<0.0001), with severe illness primarily observed in older adults (median age: 55 years). Comorbidity prevalence was notable in 41.2% of participants (n=115), dominated by hypertension (22.2%), followed by diabetes (7.5%) and chronic liver disease (5.4%). Of note, comorbidity profiles and treatment regimens were balanced across severity groups and did not differ significantly between patients with and without lymphopenia. Patients were stratified into mild, moderate, and severe severity groups and followed for a median of 50 days post-symptom onset (IQR 48 days; range 42–53 days). Full demographic and clinical details are summarized in **Table 1**.

**Table 1:** Characteristics of enrolled patients at admission.

Variables	All (N = 279)	Mild (N = 13)	Moderate (N = 218)	Severe (N = 48)	P*
Gender (male/female)	149/130	10/3	114/104	25/23	0.24
Age (year)	48(39,56)	40(25,45)	47(38,55)^a^	55(46,67)^aaaa/bbb^	<0.0001
Body temperature (°C)	37.0(36.6,37.6)	36.8(36.6,37.7)	37.0(36.5,37.5)	37.2(36.7,38.1)^b^	0.053
Systolic pressure (mmHg)	128.0(120.0,140.0)	134.0(120.5,144.5)	128.0(120.0,140.0)	128.0(121.0,136.5)	0.91
Diastolic pressure (mmHg)	82.0(75.0,89.0)	79.0(73.5,94.0)	82.0(75.0,89.3)	80.0(72.3,85.8)	0.48
Pre-existing disorders (yes/no)					
Chronic heart disease	5/274	0/13	4/214	1/47	1.0
Diabetes	21/258	1/12	16/202	4/44	0.91
Hypertention	62/217	1/12	37/181	24/24^aa/bbbb^	<0.0001
Chronic renal disease	1/278	0/13	1/217	0/48	1.0
Cancer	1/278	0/13	0/218	1/47	0.22
Chronic liver disease	15/264	0/13	12/206	3/45	0.88
HBV	2/277	0/13	2/216	0/48	1.0
Chronic lung disease	8/271	0/13	6/212	2/46	0.75
Symptoms (yes/no)					
Fever	179/100	858	136/82	35/13	0.071
Dry cough	97/182	4/9	79/139	14/34	0.63
Fatigue	89/190	5/8	71/147	13/35	0.65
Sore throat	41/238	3/10	32/186	6/42	0.53
Runny nose	19/260	1/12	17/201	1/47	0.34
Sputum production	157/122	4/9	116/102	37/11^aa/bb^	0.001
Dizzy or headache	30/249	0/13	21/197	9/39	0.11
Nausea or vomiting	31/248	0/13	22/196	9/39	0.13
Myalgia	22/257	2/11	17/201	3/45	0.49
Poor apprtite	14/265	0/13	13/205	1/47	0.63
Diarrhea	52/227	2/11	40/178	10/38	0.91
Post-symptom onset (days)	48.0(42.0,53.0)	43.0(39.5,57.5)	48.0(42.0,52.0)	50.0(43.0,55.0)	0.50

Results are presented as median (interquartile range).

*P values are the results of Kruskal-Wallis tests for continuous variables and Pearson χ^2^ tests or Fisher’s exact test for categorical variables.

^a^p values indicate a significant difference compared with the mild group:a < 0.05, aa < 0.01, aaa < 0.001, aaaa < 0.0001.

^b^p values indicate a significant difference compared with the moderate group:b < 0.05, bb < 0.01, bbb < 0.001, bbbb < 0.0001.

### Restoration of inflammatory and immune parameters during SARS-CoV-2 convalescence

**Figure 2:**
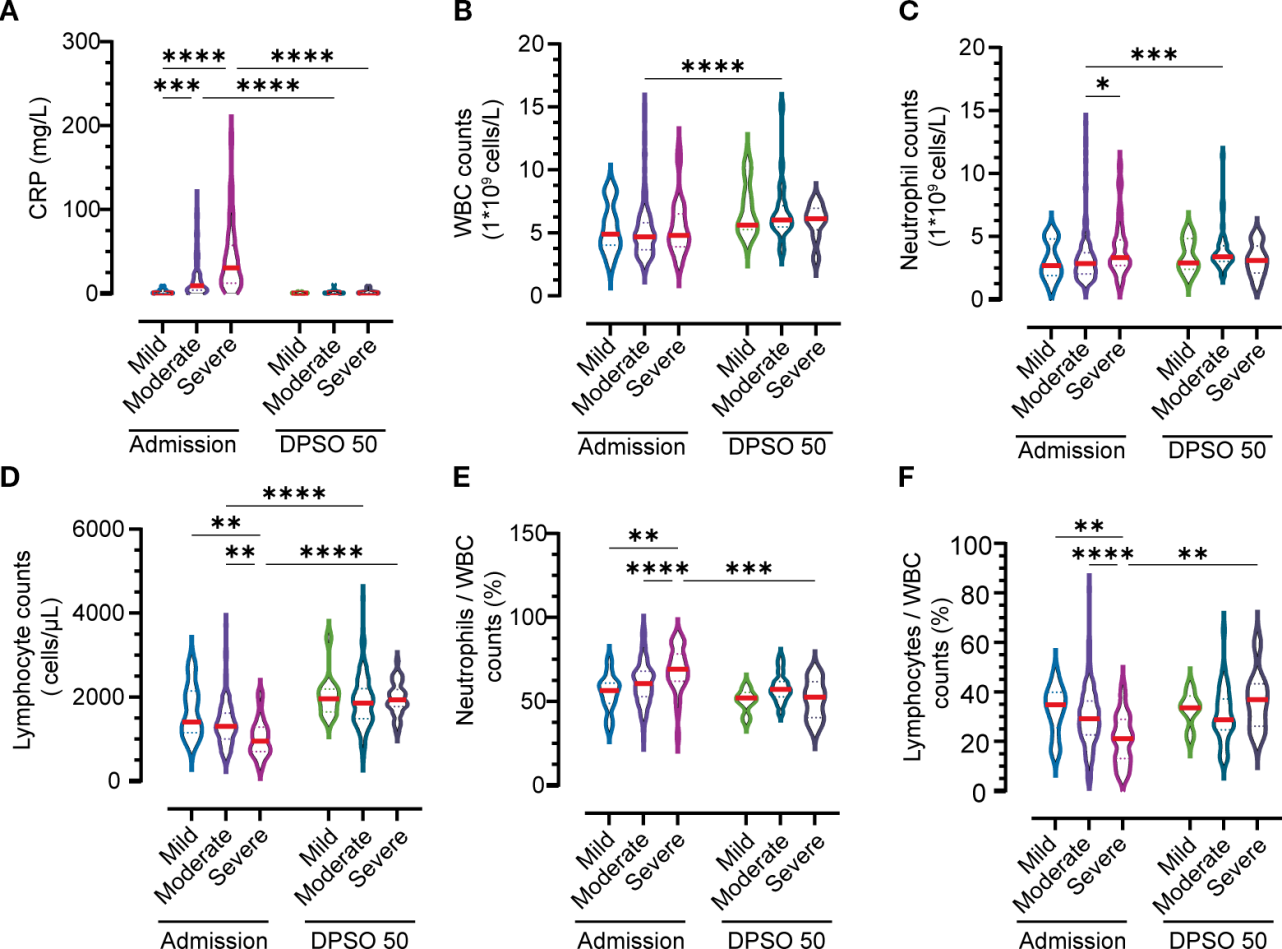
Temporal Immuno-inflammatory Dynamics Across Disease Severity Groups at Admission and 50 Days Post-Symptom Onset (DPSO50). **(A)** C-Reactive Protein (CRP) Kinetics. **(B)** White Blood Cell (WBC) Trajectories. **(C)** Neutrophil Dynamics. **(D)** Lymphocyte Recovery. **(E, F)** Neutrophil and lymphocyte subpopulation shifts in peripheral WBC composition. Comparisons employed two-way nonparametric ANOVA followed by Kruskal-Wallis *post-hoc* testing. Significant differences are indicated by *p<0.05, **p<0.01, ***p<0.001, and ****p<0.0001.

In mild cases, C-reactive protein (CRP) levels remained low throughout both the acute and convalescent phases (**Figure 2A**). However, moderate-to-severe cases exhibited elevated CRP concentrations during acute infection, which subsequently normalized during recovery. White blood cell (WBC) dynamics, composed of neutrophils and lymphocytes, revealed distinct recovery trajectories: while acute-phase inflammation suppressed total WBC and immune cell counts, these parameters recovered sufficiently by convalescence. Consequently, no statistically significant differences in WBC or neutrophil counts were observed across disease severity groups (mild, moderate, severe) in either acute or convalescent stages (**Figures 2B, C**).

Lymphocytopenia, defined by reduced lymphocyte counts during acute illness, fully resolved in moderate and severe cases by convalescence (**Figure 2D**). Notably, severe cases displayed heightened neutrophil frequencies compared to both convalescent patients at 50 days post-symptom onset (DPSO 50) and mild cases during the acute phase (**Figure 2E**). Conversely, lymphocyte depletion was most pronounced in severe acute cases but gradually rebounded to supranormal levels during recovery, surpassing baseline ranges (**Figure 2F**). These findings underscore the dynamic immune reconstitution process, with severe cases exhibiting prolonged neutrophilic inflammation and delayed lymphoid recovery despite overall parameter normalization.

### Recovery of lymphocyte subtypes during convalescence in moderate and severe cases

**Table 2** highlights incomplete immunophenotypic recovery of lymphocyte subsets in moderate and severe COVID-19 patients during convalescence. While total lymphocyte counts returned to normal ranges during the recovery phase, significant heterogeneity was observed in the restoration of specific lymphocyte subpopulations. Persistent immunophenotypic deficits were identified in critical immune cell subsets of moderate and severe cases, with notable disparities across disease severity categories.

**Table 2:** Dynamic profiles of inflammatory, immune, and cellular indicators among different groups at admission and DPSO50.

Variables	Reference ranges	Mild(N = 13)		P	Moderate(N = 218)		P	Severe(N = 48)		P
		On admission	DPSO 50		On admission	DPSO 50		On admission	DPSO 50	
CRP, mg/L	≤10	0.70(0.50,3.78)	0.50(0.50,1.20)	0.42	8.95(3.97,23.86)	1.00(0.50,1.90)	<0.0001	30.70(12.41,57.40)	0.70(0.56,4.06)	<0.0001
≥10 mg/L		0/13(0)	0/5(0)	1.0	104/214(48.6)	1/46(2.2)	<0.0001	38/47(80.9)	0/9(0)	<0.0001
WBC count, ×10^9^/L	4-10	4.90(4.02,7.11)	5.62(5.28,8.87)	0.20	4.70(3.66,5.83)	6.22(5.40,6.85)	<0.0001	4.80(3.90,6.51)	6.12(5.25,6.96)	0.16
<4 ×10^9/^L		2/13(15.4)	0/5(0)	1.0	70/213(32.9)	3/54(5.6)	<0.0001	12/47(25.5)	1/9(11.1)	0.67
>10 ×10^9/^L		0/13(0)	1/5(20.0)	1.0	8/213(3.8)	2/54(3.7)	1.0	3/47(6.4)	0/9(0)	1.0
Neutrophil count, ×10^9^/L	1.8-6.3	2.70(1.90,4.80)	2.90(2.39,4.85)	0.63	2.82(2.01,3.70)	3.40(3.02,4.27)	<0.0001	3.32(2.70,4.72)	3.10(2.11,4.25)	0.38
<1.8 ×10^9^/L		3/13(23.1)	0/5(0)	0.52	39/212(18.4)	0/53(0)	<0.0001	4/46(8.7)	1/9(11.1)	1.0
>6.3 ×10^9^/L		0/13(0)	0/5(0)	1.0	9/212(4.3)	3/53(5.7)	0.71	5/46(10.9)	0/9(0)	0.58
Lymphocytes, cells/μL	1100–3200	1410(1150,2145)	1959(1648,2194)	0.11	1300(1000,1610)	1861(1486,2203)	<0.0001	950(700,1285)	1971(1793,2181)	<0.0001
<1100 cells/μL		2/13(15.4)	0/13(0)	0.48	58/213(27.2)	13/218(6.0)	<0.0001	27/46(58.7)	0/47(0)	<0.0001
NLR		1.57(1.23,2.41)	1.49(1.22,2.07)	0.62	2.08(1.45,2.97)	1.87(1.48,2.50)	0.29	3.22(2.25,6.08)	1.29(0.86,2.02)	<0.0001
Neutropil/WBC,%		56.5(48.8,60.9)	52.1(44.7,55.3)	0.25	60.5(52.8,67.6)	57.1(52.7,61.9)	0.11	69.2(62.1,78.2)	52.5(40.3,61.7)	0.0002
Lymphocyte/WBC,%		34.9(25.5,39.8)	33.6(27.8,38.3)	0.92	29.2(22.4,36.4)	29.0(24.7,37.3)	0.49	21.1(13.1,28.9)	36.9(26.1,43.3)	0.0003
Platelets, ×10^9^/L	100-300	192(174,217)	225(185,262)	0.21	188(150,242)	250(209,292)	<0.0001	182(147,217)	231(193,311)	0.046
<100×10^9^/L		0/13(0)	0/5(0)	1.0	8/213(3.8)	0/53(0)	0.36	0/47(0)	0/9(0)	1.0
>300×10^9^/L		0/13(0)	0/5(0)	1.0	1/213(0.5)	12/53(22.6)	<0.0001	3/47(6.4)	2/9(22.2)	0.18
B cell, cells/μL	90–560	266(121,480)	238(183,314)	0.82	146(94,219)	186(139,254)	0.004	169(90,263)	179(131,230)	0.94
<90 cells/μL		0/7(0)	0/13(0)	1.0	10/42(23.8)	16/218(7.3)	0.003	2/8(25.0)	6/48(12.5)	0.32
B cell %		14.0(8.6,32.8)	12.6(9.3,16.0)	0.73	11.6(8.8,20.7)	10.9(8.0,14.1)	0.042	22.8(13.4,39.2)	9.3(6.7,11.1)	0.0005
CD4 T cell, cells/μL	414-1123	529(439,771)	725(585,815)	0.14	547(397,749)	658(510,830)	0.0007	448(230,510)	712(566,821)	<0.0001
<400 cells/μL		1/8(12.5)	0/13(0)	1.0	21/84(25.0)	21/218(9.6)	0.001	7/16(43.8)	2/47(4.3)	0.001
CD4 T cell %		35.7(33.5,46.8)	37.0(32.7,40.6)	0.52	41.9(34.9,50.0)	36.8(32.1,42.2)	<0.0001	36.2(29.9,54.9)	34.3(30.5,40.6)	0.25
CD8 T cell, cells/μL	238-874	398(289,527)	434(406,669)	0.21	325(234,433)	474(371,609)	<0.0001	209(151,495)	535(409,730)	<0.0001
<220 cells/μL		0/8(0)	0/13(0)	1.0	17/84(20.2)	8/218(3.7)	<0.0001	8/16(50.0)	0/47(0)	<0.0001
CD8 T cell %		27.1(21.5,37.4)	26.0(23.6,28.8)	0.83	23.9(17.1,32.6)	27.1(22.8,32.3)	0.036	27.6(16.2,37.4)	28.4(22.0,35.8)	0.71
CD4/CD8 T cell ratio	1.5–2.0	1.49(1.12,1.61)	1.32(1.17,1.88)	0.68	1.79(1.18,2.50)	1.37(1.11,1.67)	<0.0001	1.72(0.97,2.42)	1.22(0.96,1.54)	0.082
<1.2		2/8(25.0)	4/13(30.8)	1.0	21/84(25.0)	73/218(33.5)	0.17	6/16(37.5)	23/47(48.9)	0.56
NK cell, cells/μL	150–1100	315(167,391)	305(228,497)	0.47	215(137,409)	331(205,475)	0.0006	241(129,300)	384(255,587)	0.037
<150 cells/μL		0/7(0)	1/13(7.7)	0.52	12/41(29.3)	17/218(7.8)	<0.0001	2/8(25.0)	4/48(8.3)	0.15
NK cell %		27.9(9.1,35.0)	18.0(11.6,26.2)	0.49	16.5(12.1,29.0)	18.3(12.6,25.6)	0.72	24.7(19.1,41.7)	20.8(13.1,29.6)	0.14

The data are presented as median and interquartile range (IQR), with n/N (%) representing the number of observations in each group.

P values are the results of Mann-Whitney U test for continuous variables and Pearson χ^2^ tests or Fisher’s exact test for categorical variables.

DPSO 50, Days post-symptom onset. CRP, C-reactive protein. WBC, White blood cell; NLR, neutrophil to lymphocyte ratio; NK cells, natural killer cells.

First, B cells showed incomplete recovery in 7.3% of moderate cases and 12.5% of severe cases, remaining below the baseline threshold level (BTL; defined as ≥1.0×10^9^/L). CD4^+^ T cells exhibited deficits in 9.6% of moderate cases, yet only 4.3% of severe cases fell below BTL—a finding suggesting severity-dependent resilience in this subset. In contrast, CD8^+^ T cells demonstrated full recovery in severe cases, whereas moderate cases retained subthreshold levels in 3.7% of patients. NK cells, however, remained impaired in both groups, with 7.8% (moderate) and 8.3% (severe) of patients below BTL.

Notably, mild cases demonstrated full restoration of all lymphocyte subsets, exceeding BTL thresholds across all measured subtypes. These findings underscore severity-dependent immune reconstitution failure, particularly in B and NK cell recovery. This partial restoration may perpetuate prolonged immunocompromise in severe infections, increasing susceptibility to secondary infections or impaired long-term immune memory.

### Total T lymphocyte and subpopulation dynamics in persistent B-cell lymphopenia during SARS-CoV-2 convalescence

**Figure 3:**
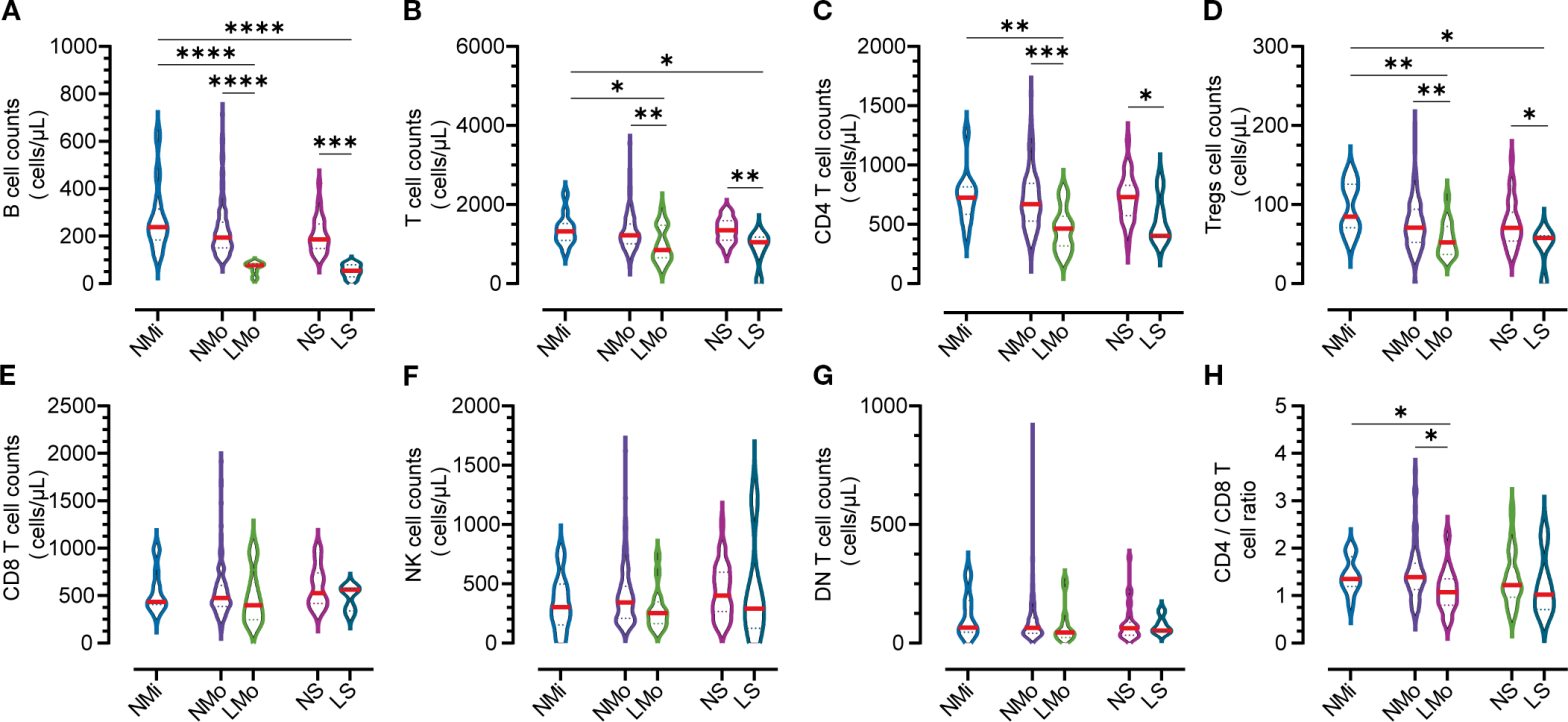
Counts of lymphocyte subtypes in groups with normal and low B cell counts on DPSO50. **(A)** B cell Lymphopenia: Moderate and severe groups exhibited B cell lymphopenia. **(B)** Total T Cell count. **(C)** CD4 T Cell count. **(D)** Tregs count: The counts of total T cells, CD4 T cells, and Tregs were decreased in the B cell lymphopenia group. **(E)** CD8 T Cell count. **(F)** NK cell count. **(G)** DN T Cell count: There were no significant differences in the counts of CD8 T cells, NK cells, and DN T cells among the five groups. **(H)** CD4/CD8 T Cell ratio: The CD4/CD8 T cell ratio in the low B cell count group (LMo) was lower than that in the normal B cell count group (NMo). NMi: Mild cases with normal B cell counts. NMo: Moderate cases with normal B cell counts. LMo: Moderate cases with B cell lymphopenia. NS: Severe cases with normal B cell counts. LS: Severe cases with B cell lymphopenia. Statistical analysis was performed using a two-way non-parametric ANOVA, followed by a non-parametric Kruskal-Wallis multiple comparisons test. Statistical significance is denoted by *p<0.05, **p<0.01, ***p<0.001, and ****p<0.0001.

During convalescence, around 90.4-97.9% of COVID-19 patients in moderate and severe cases respectively had their total T lymphocyte counts return to the normal range (**Table 3**). Moderate (LMo) and severe (LS) cases with BTL exhibited significantly reduced total T cell, CD4 T cell, and regulatory T cell (Treg) counts compared to non-BTL counterparts (NMo and NS groups at DPSO 50) (**Figures 3B-D**). **Table 3** illustrates that the rates of lymphocyte subtype cell counts in cases with BTL were 50.0% and 16.7% for T-cell counts, 43.8% and 20.0% for CD4 T cell counts, 18.8% and 0% for CD8 T cell counts, and 18.8% and 16.7% for NK cells in the LMo and LS groups, respectively. There were no differences in CD8 T cell, NK cell, and DN T cell counts among the groups during convalescence (**Figures 3E-G**). The LMo group exhibited a lower CD4/CD8 T cell ratio compared to the NMo group, as shown in **Figure 3H**.

**Table 3:** Lymphocyte and its subtype cell profiles in convalescent patients with or without B cell lymphocytopenia in mild, moderate, and severe COVID-19.

Variables	NMi (N = 13)	Moderate-All (N = 218)	NMo (N = 202)	LMo (N = 16)	P*	Severe-All (N = 48)	NS (N = 42)	LS (N = 6)	P*	P^ǂ^
Lymphocytes, cells/μL	1959(1648,2194)	1861(1486,2203)	1897(1515,2245)	1264(907,1831)^a/bb^	<0.0001	1971(1793,2181)	1988(1813,2219)	1400(1250,2114)	0.053	0.0004
<1100,cells/μL	0/13(0)	13/218(6.0)	7/202(3.5)	6/16(37.5)	<0.0001	0/47(0)	0/42(0)	0/5(0)	1.0	**-**
B cell, cells/μL	238(184,314)	186(139,254)	194(152,259)	77(52,84)^aaaa/bbbb^	<0.0001	179(131,230)	187(148,252)	55(28,79)^aaa/bbb/cc^	<0.0001	<0.0001
<90 cells/μL	0/13(0)	15/218(6.9)	0/202(0)	16/16(100)^aaaa^	<0.0001	6/48(12.5)	0/42(0)	6/6(100)^aaaa^	<0.0001	–
B cell %	12.6(9.3,16.0)	10.9(8.0,14.1)	11.1(8.2,14.2)	4.9(3.0,7.9)	<0.0001	9.3(6.7,11.1)	9.9(8.4,11.7)	4.5(2.8,5.4)	<0.0001	0.043
Total T lymphocyte count,cells/μL	1319(1090,1517)	1207(983,1503)	1221(1009,1511)	846(650,1477)	0.006	1299(1074,1554)	1346(1095,1593)	1046(604,1174)	0.009	0.004
<770 cells/μL	0/13(0)	21/218(9.6)	13/202(6.4)	8/16(50.0)^aa^	<0.0001	1/48(2.1)	0/42(0)	1/6(16.7)	0.13	–
CD4 T cell, cells/μL	725(585,815)	658(510,830)	669(531,848)	464(318,567)^a/bbb^	<0.0001	712(566,821)	728(573,829)	402(399,713)	0.041	0.0002
<400 cells/μL	1/13(7.7)	21/218(9.6)	14/202(6.9)	7/16(43.8)^a^	<0.0001	2/47(4.3)	1/42(2.4)	1/5(20.0)	0.20	–
CD4 T cell %	37.0(31.9,40.0)	36.8(32.1,42.2)	36.8(32.2,42.3)	34.2(29.8,39.3)	0.14	34.3(30.5,40.6)	34.8(30.7,40.8)	33.0(27.1,37.9)	0.41	0.24
CD8 T cell, cells/μL	434(406,669)	474(371,609)	476(387,609)	399(247,639)	0.19	535(409,730)	527(418,740)	564(340,576)	0.30	0.16
<220 cells/μL	1/13(7.7)	8/218(3.7)	5/202(2.5)	3/16(18.8)	0.015	0/47(0)	0/42(0)	0/5(0)	1.0	–
CD8 T cell %	26.0(22.6,28.6)	27.1(22.8,32.3)	26.8(22.4,31.5)	32.5(27.6,38.5)	0.005	28.4(22.0,35.8)	28.0(21.9,35.7)	34.6(20.5,41.7)	0.49	0.044
CD4/CD8 T cell ratio	1.32(1.17,1.88)	1.37(1.11,1.67)	1.39(1.13,1.68)	1.07(0.80,1.35)^b^	0.004	1.22(0.96,1.54)	1.22(0.97,1.61)	1.02(0.71,1.78)	0.46	0.022
<1.2	4/13(30.8)	73/218(33.5)	63/202(31.2)	10/16(62.5)	0.024	23/47(48.9)	20/42(47.6)	3/5(60.0)	0.67	–
NK cell, cells/μL	305(228,497)	331(205,475)	343(208,480)	253(164,347)	0.028	381(244,583)	401(265,598)	291(124,654)	0.35	0.089
<150 cells/μL	1/13(7.7)	17/218(7.8)	14/202(6.9)	3/16(18.8)	0.12	4/48(8.3)	3/42(7.1)	1/6(16.7)	0.43	–
NK cells %	18.0(11.6,26.2)	18.3(12.6,25.6)	18.2(12.4,25.6)	20.0(14.7,24.5)	0.56	20.8(13.1,29.6)	19.9(12.7,29.9)	28.0(15.3,37.7)	0.36	0.55
DN T cell, cells/μL	65.2(45.4,178.9)	60.9(39.0,105.8)	64.0(41.0,106.0)	44.9(23.3,83.0)	0.071	62.4(34.5,85.9)	62.8(33.1,88.0)	52.0(49.3,108.6)	0.67	0.29
DN T %	6.1(3.2,14.7)	4.9(3.5,8.4)	4.9(3.6,8.4)	4.8(2.9,11.9)	0.73	4.2(3.2,6.4)	4.2(2.9,6.0)	4.7(4.4,11.5)	0.15	0.37
Tregs, cells/μL	84.6(70.7,125.7)	69.8(51.7,92.4)	70.9(52.1,94.4)	52.1(36.8,72.2)^aa^	0.006	66.4(52.7,86.6)	70.3(53.9,90.0)	57.6(38.4,60.5)^a^	0.044	0.002
Tregs %	7.1(5.5,8.9)	5.9(5.0,6.9)	5.9(5.0,6.8)	5.8(5.2,7.4)	0.61	5.6(4.1,6.8)	5.5(4.1,6.9)	5.8(3.3,6.6)	0.78	0.11

The data are presented as median and interquartile range (IQR), with n/N (%) representing the number of observations in each group.

*P indicates values are the result of Mann-Whitney U test for continuous variables and Pearson χ^2^ tests or Fisher’s exact test for categorical variables.

^ǂ^P values are the result of Kruskal-Wallis tests for continuous variables among NMi, NMo, LMo, NS and LS.

^a^p values indicate a significant difference compared with NMi; a < 0.05, aa < 0.01, aaa < 0.001, aaaa < 0.0001.

^b^p values indicate a significant difference compared with NMo; b < 0.05, bb < 0.01, bbb < 0.001,bbbb < 0.0001.

^c^p values indicate a significant difference compared with NS; c < 0.05,cc < 0.01.

NMi, mild cases with B cell count in the normal range; NMo, moderate cases with B cell count in the normal range; LMo, moderate cases with B-cell lymphopenia; NS, severe cases with B cell count in the normal range; LS, severe cases with B-cell lymphopenia; DN T, double negative T cells; Tregs, regulatory T cells.

### Correlation analysis of Tregs counts with B cell and CD4^+^ T cell counts

**Figure 4:**
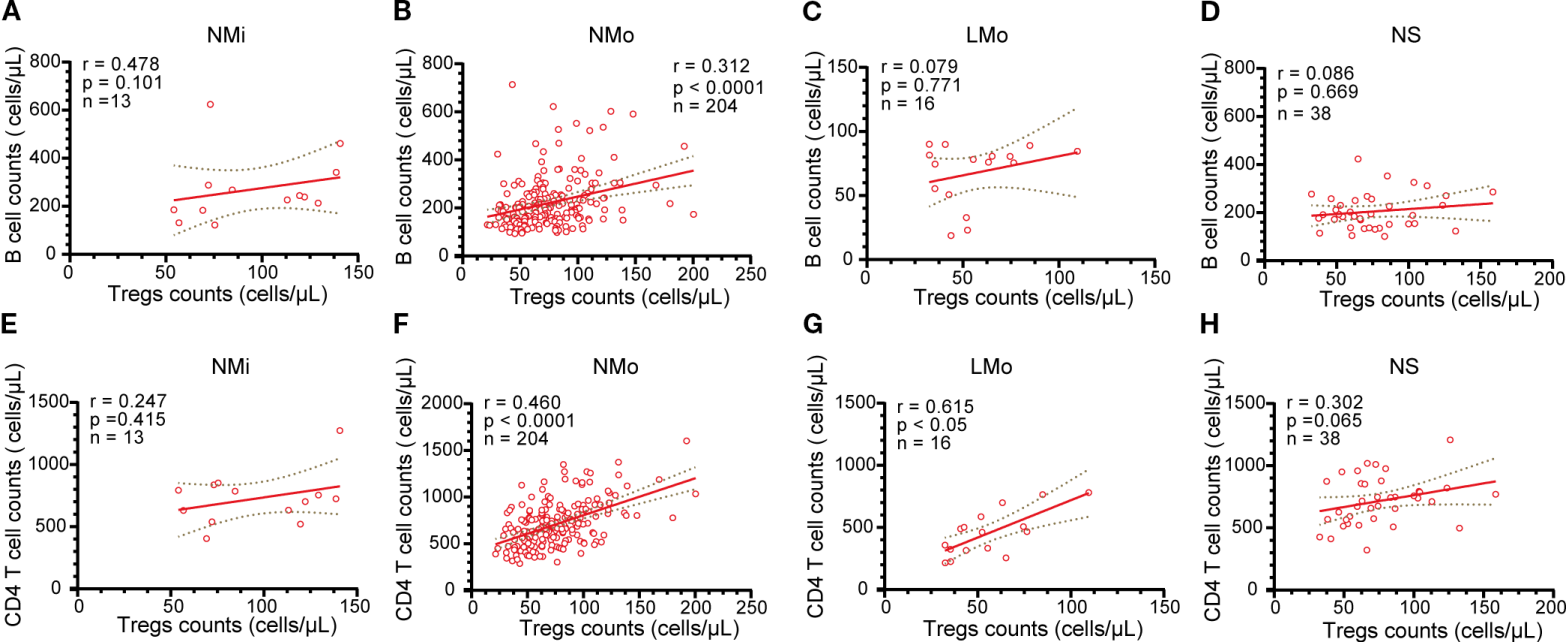
Analysis of the relationship between B cells and Tregs cells, as well as CD4 T cells and Tregs in NMi, NMo, LMo, and NS groups at DPSO50. **(A-D)** Correlation analysis between B cell counts and Tregs counts in NMi, NMo, LMo, and NS groups, respectively. **(E-H)** Correlation analysis between T cell counts and Tregs counts in NMi, NMo, LMo, and NS groups, respectively. Pearson correlation analysis was used to determine the correlations.

In the NMi group, the counts of Tregs and B cells demonstrated a positive correlation; however, the correlation was not statistically significant (r=0.478, n=13, p > 0.05, **Figure 4A**), likely due to the smaller sample size. Conversely, in the NMo group, a significant positive correlation was found between the counts of Tregs and B cells (r=0.312, n=204, p<0.0001, **Figure 4B**). However, no significant correlations were observed between Tregs counts and B cell counts in the LMo and NS groups (r=0.079, n=16 and r=0.086, n=38, respectively, both p>0.05, **Figures 4C, D**).

Both the NMo and LMo groups displayed positive correlations between the counts of Tregs and CD4 T cells (r=0.460, n=204, and r=0.615, n=16, p<0.0001 and p<0.05, respectively, **Figures 4F, G**). Conversely, no significant correlations were found between Tregs counts and CD4 T cell counts in the NMi and NS groups (r=0.247, n=13 and r=0.302, n=38, respectively, both p>0.05, **Figures 4E, H**).

## Discussion

This study investigated immune response dynamics in unvaccinated individuals with mild, moderate, and severe SARS-CoV-2 infections, focusing on longitudinal immune cell profiling to elucidate immunopathological mechanisms underlying prolonged recovery. Key findings revealed persistent B cell lymphopenia (<90 cells/µL) at 50 days post-symptom onset (DPSO 50) in 7.3% of moderate and 12.5% of severe cases, despite clinical improvement, suggesting unresolved immune dysfunction. This deficiency correlated with reduced serum serotonin levels (Wong et al., 2023)—a critical mediator of B cell proliferation (Iken et al., 1995)—highlighting potential immune axis dysregulation in Long COVID pathogenesis.

Further analysis demonstrated that B cell lymphopenia coincided with significant reductions in total T cells, CD4 T cells, and regulatory T cells (Tregs) during convalescence, particularly in moderate cases (LMo), where CD4/CD8 T cell ratios fell below normal thresholds. While severe cases (LS) exhibited similar trends, sample size limitations precluded statistical significance. These observations align with serotonin’s role in T cell proliferation via 5-HT1A receptor activation (Aune et al., 1993; Iken et al., 1995; Abdouh et al., 2001), suggesting that serotonin depletion in Long COVID (Wong et al., 2023) may selectively impair CD4 T and Treg subsets while sparing CD8 T cells.

Notably, the strong positive correlation between CD4 T cells and Tregs in moderate cases (LMo) disappeared in severe convalescent patients (LS), potentially reflecting Treg migration to inflamed tissues, exhaustion, or reduced peripheral availability (Miedema et al., 2023). This loss of Treg/CD4 T cell coordination is clinically consequential, as Treg deficiency exacerbates inflammation and impairs Th17/Treg balance—a dysregulation linked to respiratory failure and poor Long COVID outcomes (Dhawan et al., 2023). Given Tregs’ role in immune homeostasis and autoimmunity prevention (Cheru et al., 2023), their depletion may perpetuate chronic inflammation and autoantibody production in post-acute sequelae.

The combined immunological profile—persistent B cell lymphopenia, CD4 T cell/Treg depletion, and disrupted CD4/CD8 ratios—defines a distinctive immunopathological signature in moderate-to-severe cases, resembling immunological patterns in HIV/EBV co-infections (Richard et al., 2010; Rosado-Sanchez et al., 2017). These biomarkers could aid in identifying patients at risk of prolonged immunocompromise, guiding targeted therapies such as Treg-boosting interventions or immune checkpoint modulation. However, study limitations—including small sample size and limited longitudinal follow-up—warrant validation through large-scale cohorts incorporating serotonin quantification and single-cell immune profiling (e.g., transcriptomic analysis of exhausted T cells).

Several limitations of this study should be acknowledged. First, the sample size in certain subgroups, particularly those with persistent B-cell lymphopenia (LMo: n = 16; LS: n = 6), was relatively small, which may limit the statistical power and generalizability of correlation analyses, such as those involving Treg subsets. Second, the follow-up period was limited to 50 days post-symptom onset, which may not fully capture long-term immune reconstitution trajectories or the complete spectrum of post-acute sequelae. Third, while this study focused on phenotypic immune profiling, it did not include functional assays, such as cytokine production or antigen-specific responses, leaving the functional competence of altered lymphocyte subsets unclear. Furthermore, although discussed in the context of emerging literature, the proposed link between immune dysregulation and serotonin deficiency remains speculative; serotonin levels were not measured in this study, and this hypothesis warrants further validation. Finally, correlation analyses were exploratory in nature and were not adjusted for multiple testing, a factor that should be considered when interpreting the results. Future studies involving larger cohorts, extended follow-up durations, functional validations, and integrated multi-omics approaches are needed to confirm and extend these findings.

In conclusion, this work identifies persistent defects in the B cell/T cell axis as a critical immunological hallmark of Long COVID. These alterations provide potential biomarkers for immunopathological stratification and therapeutic targeting, while also underscoring the heightened risk of infection among immunocompromised survivors, who may benefit from tailored preventive strategies.

## Data Availability

The original contributions presented in the study are included in the article/supplementary material. Further inquiries can be directed to the corresponding author.

## References

[B1] Abdouh M. Storring J. M. Riad M. Paquette Y. Albert P. R. Drobetsky E.. (2001). Transcriptional mechanisms for induction of 5-HT1A receptor mRNA and protein in activated B and T lymphocytes. J. Biol. Chem. 276, 4382–4388. doi: 10.1074/jbc.M004559200, PMID: 11080494

[B2] Alsalman A. Al-Mterin M. A. Elkord E. (2022). Role of T regulatory cells and myeloid-derived suppressor cells in COVID-19. J. Immunol. Res. 2022, 5545319. doi: 10.1155/2022/5545319, PMID: 35497875 PMC9042623

[B3] Altmann D. M. Whettlock E. M. Liu S. Arachchillage D. J. Boyton R. J. (2023). The immunology of long COVID. Nat. Rev. Immunol. 23, 618–634. doi: 10.1038/s41577-023-00904-7, PMID: 37433988

[B4] An H. Zhang J. Li T. Hu Y. Wang Q. Chen C.. (2022). Inflammation/coagulopathy/immunology responsive index predicts poor COVID-19 prognosis. Front. In Cell. Infect. Microbiol. 12, 807332. doi: 10.3389/fcimb.2022.807332, PMID: 35310845 PMC8930906

[B5] Apoil P. A. Puissant-Lubrano B. Congy-Jolivet N. Peres M. Tkaczuk J. Roubinet F.. (2017). Reference values for T, B and NK human lymphocyte subpopulations in adults. Data Brief. 12, 400–404. doi: 10.1016/j.dib.2017.04.019, PMID: 28491945 PMC5415546

[B6] Aune T. M. McGrath K. M. Sarr T. Bombara M. P. Kelley K. A. (1993). Expression of 5HT1a receptors on activated human T cells. Regulation of cyclic AMP levels and T cell proliferation by 5-hydroxytryptamine. J. Immunol. 151, 1175–1183. doi: 10.4049/jimmunol.151.3.1175, PMID: 8393041

[B7] Chen G. Wu D. Guo W. Cao Y. Huang D. Wang H.. (2020). Clinical and immunological features of severe and moderate coronavirus disease 2019. J. Clin. Invest. 130, 2620–2629. doi: 10.1172/JCI137244, PMID: 32217835 PMC7190990

[B8] Cheru N. Hafler D. A. Sumida T. S. (2023). Regulatory T cells in peripheral tissue tolerance and diseases. Front. Immunol. 14, 1154575. doi: 10.3389/fimmu.2023.1154575, PMID: 37197653 PMC10183596

[B9] Dhawan M. Rabaan A. A. Alwarthan S. Alhajri M. Halwani M. A. Alshengeti A.. (2023). Regulatory T cells (Tregs) and COVID-19: unveiling the mechanisms, and therapeutic potentialities with a special focus on long COVID. Vaccines (Basel) 11 (3), 699. doi: 10.3390/vaccines11030699, PMID: 36992283 PMC10059134

[B10] Guan W.-J. Ni Z.-Y. Hu Y. Liang W.-H. Ou C.-Q. He J.-X.. (2020). Clinical characteristics of coronavirus disease 2019 in China. New Engl. J. Med. 382, 1708–1720. doi: 10.1056/NEJMoa2002032, PMID: 32109013 PMC7092819

[B11] Huang C. Wang Y. Li X. Ren L. Zhao J. Hu Y.. (2020). Clinical features of patients infected with 2019 novel coronavirus in Wuhan, China. Lancet 395, 497–506. doi: 10.1016/S0140-6736(20)30183-5, PMID: 31986264 PMC7159299

[B12] Iken K. Chheng S. Fargin A. Goulet A. C. Kouassi E. (1995). Serotonin upregulates mitogen-stimulated B lymphocyte proliferation through 5-HT1A receptors. Cell Immunol. 163, 1–9. doi: 10.1006/cimm.1995.1092, PMID: 7758118

[B13] Jiang Y. Wei X. Guan J. Qin S. Wang Z. Lu H.. (2020). COVID-19 pneumonia: CD8(+) T and NK cells are decreased in number but compensatory increased in cytotoxic potential. Clin. Immunol. 218, 108516. doi: 10.1016/j.clim.2020.108516, PMID: 32574709 PMC7305921

[B14] Jin S. An H. Zhou T. Li T. Xie M. Chen S.. (2021). Sex- and age-specific clinical and immunological features of coronavirus disease 2019. PloS Pathogens 17, e1009420. doi: 10.1371/journal.ppat.1009420, PMID: 33770147 PMC8026060

[B15] Jin X. H. Zhou H. L. Chen L. L. Wang G. F. Han Q. Y. Zhang J. G.. (2021). Peripheral immunological features of COVID-19 patients in Taizhou, China: A retrospective study. Clin. Immunol. 222, 108642. doi: 10.1016/j.clim.2020.108642, PMID: 33253854 PMC7695552

[B16] Kam K. M. Leung W. L. Kwok M. Y. Hung M. Y. Lee S. S. Mak W. P. (1996). Lymphocyte subpopulation reference ranges for monitoring human immunodeficiency virus-infected Chinese adults. Clin. Diagn. Lab. Immunol. 3, 326–330. doi: 10.1128/cdli.3.3.326-330.1996, PMID: 8705678 PMC170341

[B17] Kostopoulos I. V. Orologas-Stavrou N. Rousakis P. Panteli C. Ntanasis-Stathopoulos I. Charitaki I.. (2021). Recovery of innate immune cells and persisting alterations in adaptive immunity in the peripheral blood of convalescent plasma donors at eight months post SARS-CoV-2 infection. Microorganisms 9 (3), 546. doi: 10.3390/microorganisms9030546, PMID: 33800807 PMC8000115

[B18] Kudryavtsev I. V. Arsentieva N. A. Korobova Z. R. Isakov D. V. Rubinstein A. A. Batsunov O. K.. (2022). Heterogenous CD8+ T cell maturation and 'Polarization' in acute and convalescent COVID-19 patients. Viruses 14 (9), 1906. doi: 10.3390/v14091906, PMID: 36146713 PMC9504186

[B19] Miedema J. R. de Jong L. J. van Uden D. Bergen I. M. Kool M. Broos C. E.. (2023). Circulating T cells in sarcoidosis have an aberrantly activated phenotype that correlates with disease outcome. J. Autoimmun. 149, 103120. doi: 10.1016/j.jaut.2023.103120, PMID: 37863732

[B20] Orologas-Stavrou N. Politou M. Rousakis P. Kostopoulos I. V. Ntanasis-Stathopoulos I. Jahaj E.. (2020). Peripheral blood immune profiling of convalescent plasma donors reveals alterations in specific immune subpopulations even at 2 months post SARS-CoV-2 infection. Viruses 13 (1), 26. doi: 10.3390/v13010026, PMID: 33375675 PMC7824046

[B21] Richard Y. Amiel C. Jeantils V. Mestivier D. Portier A. Dhello G.. (2010). Changes in blood B cell phenotypes and Epstein-Barr virus load in chronically human immunodeficiency virus-infected patients before and after antiretroviral therapy. J. Infect. Dis. 202, 1424–1434. doi: 10.1086/656479, PMID: 20874514

[B22] Rosado-Sanchez I. Herrero-Fernandez I. Alvarez-Rios A. I. Genebat M. Abad-Carrillo M. A. Ruiz-Mateos E.. (2017). A lower baseline CD4/CD8 T-cell ratio is independently associated with immunodiscordant response to antiretroviral therapy in HIV-infected subjects. Antimicrob. Agents Chemother. 61 (8), e00605-17. doi: 10.1128/AAC.00605-17, PMID: 28559274 PMC5527653

[B23] Rudolph A. E. Al Akoury N. Bogdanenko N. Markus K. Whittle I. Wright O.. (2025). Factors affecting the impact of COVID-19 vaccination on post COVID-19 conditions among adults: A systematic literature review. Hum. Vaccin. Immunother. 21, 2474772. doi: 10.1080/21645515.2025.2474772, PMID: 40079963 PMC11913386

[B24] Ryan F. J. Hope C. M. Masavuli M. G. Lynn M. A. Mekonnen Z. A. Yeow A. E. L.. (2022). Long-term perturbation of the peripheral immune system months after SARS-CoV-2 infection. BMC Med. 20, 26. doi: 10.1186/s12916-021-02228-6, PMID: 35027067 PMC8758383

[B25] Sherina N. Piralla A. Du L. Wan H. Kumagai-Braesch M. Andréll J.. (2021). Persistence of SARS-CoV-2-specific B and T cell responses in convalescent COVID-19 patients 6–8 months after the infection. Med. (New York NY) 2, 281–295.e4. doi: 10.1016/j.medj.2021.02.001, PMID: 33589885 PMC7874960

[B26] Shuwa H. A. Shaw T. N. Knight S. B. Wemyss K. McClure F. A. Pearmain L.. (2021). Alterations in T and B cell function persist in convalescent COVID-19 patients. Med 2, 720–35 e4. doi: 10.1016/j.medj.2021.03.013, PMID: 33821250 PMC8011689

[B27] Votto M. Castagnoli R. Marseglia G. L. Licari A. Brambilla I. (2023). COVID-19 and autoimmune diseases: is there a connection? Curr. Opin. Allergy Clin. Immunol. 23, 185–192. doi: 10.1097/ACI.0000000000000888, PMID: 36728317

[B28] Wong A. C. Devason A. S. Umana I. C. Cox T. O. Dohnalová L. Litichevskiy L.. (2023). Serotonin reduction in post-acute sequelae of viral infection. Cell 186 (22), 4851–4867.e20. doi: 10.1016/j.cell.2023.09.013, PMID: 37848036 PMC11227373

[B29] Yang J. Zhong M. Zhang E. Hong K. Yang Q. Zhou D.. (2021). Broad phenotypic alterations and potential dysfunction of lymphocytes in individuals clinically recovered from COVID-19. J. Mol. Cell Biol. 13, 197–209. doi: 10.1093/jmcb/mjab014, PMID: 33751111 PMC7989217

[B30] Zahran A. M. Zahran Z. A. M. Mady Y. H. Mahran E. Rashad A. Makboul A.. (2021). Differential alterations in peripheral lymphocyte subsets in COVID-19 patients: upregulation of double-positive and double-negative T cells. Multidiscip. Respir. Med. 16, 758. doi: 10.4081/mrm.2021.758, PMID: 34221400 PMC8215531

